# Association of body mass index and COPD exacerbation among patients with chronic bronchitis

**DOI:** 10.1186/s12931-022-01957-3

**Published:** 2022-03-07

**Authors:** Sun Hye Shin, Sung Ok Kwon, Victor Kim, Edwin Kepner Silverman, Tae-Hyung Kim, Deog Kyeom Kim, Yong Il Hwang, Kwang Ha Yoo, Woo Jin Kim, Hye Yun Park

**Affiliations:** 1 Division of Pulmonary and Critical Care Medicine, Department of Medicine, Samsung Medical Center, Sungkyunkwan University School of Medicine, 81 Irwon-ro, Gangnam-gu, Seoul, 06351 Republic of Korea; 2 Biomedical Research Institute, Kangwon National University Hospital, Chuncheon, Republic of Korea; 3 Lewis Katz School of Medicine at, Temple University, Philadelphia, PA USA; 4 Channing Division of Network Medicine, Division of Pulmonary and Critical Care Medicine, Department of Medicine, Brigham and Women’s Hospital and Harvard Medical School, Boston, USA; 5 Division of Pulmonology, Department of Internal Medicine, Hanyang University Guri Hospital, Hanyang University College of Medicine, Guri, Republic of Korea; 6 Division of Pulmonary and Critical Care Medicine, Department of Internal Medicine, Seoul Metropolitan Government Seoul National University Boramae Medical Center, Seoul, Republic of Korea; 7 Department of Pulmonary, Allergy and Critical Care Medicine, Hallym University Sacred Heart Hospital, Anyang, Republic of Korea; 8 Department of Internal Medicine, Konkuk University School of Medicine, Seoul, Republic of Korea; 9 Department of Internal Medicine and Environmental Health Center, Kangwon National University Hospital, Kangwon National University School of Medicine, 156 Baengyeong-ro, Chuncheon-si, Gangwon-do 200-722 Republic of Korea

**Keywords:** Body mass index, Chronic bronchitis, COPD, Exacerbation

## Abstract

**Background and objective:**

Chronic obstructive pulmonary disease (COPD) patients with a body mass index (BMI) < 25 kg/m^2^ are prone to develop adverse event of pharmacological treatment for frequent exacerbation. As chronic bronchitis (CB) is one of the strong risk factors of exacerbation, we investigated the associations between BMI and COPD exacerbations in patients with CB.

**Methods:**

Patients with COPD were included from the Korean COPD Subgroup Study (KOCOSS), a multicenter observational cohort study. CB was defined using the St. George’s Respiratory Questionnaire and the participants were categorized according to BMI cut-off of 25 kg/m^2^. Exacerbations during a 1-year follow-up were compared among four groups: non-CB with BMI ≥ 25 kg/m^2^, non-CB with BMI < 25 kg/m^2^, CB with BMI ≥ 25 kg/m^2^, and CB with BMI < 25 kg/m^2^.

**Results:**

Among the 1264 patients with COPD, 451 (35.7%) had CB and 353 (27.9%) had both CB and BMI < 25 kg/m^2^. The COPD exacerbation risk increased across the non-CB with BMI < 25 kg/m^2^, CB with BMI ≥ 25 kg/m^2^, and CB with BMI < 25 kg/m^2^ groups (adjusted incidence rate ratio [95% confidence interval] 1.21 [0.89–1.62], 1.20 [0.77–1.88], and 1.41 [1.02–1.91], respectively, compared to the non-CB with BMI ≥ 25 kg/m^2^ group).

**Conclusions:**

COPD patients having both CB and a BMI < 25 kg/m^2^ are at higher risk of exacerbations. Considering that a BMI < 25 kg/m^2^ often limits treatment options preventing exacerbations, modified guidelines might be needed for non-obese CB patients in Asia.

**Supplementary Information:**

The online version contains supplementary material available at 10.1186/s12931-022-01957-3.

## Background

Acute exacerbations of chronic obstructive pulmonary disease (COPD) are associated with prolonged detrimental effects such as a decline in lung function, reduced health-related quality of life, and worse survival [1–3]. Moreover, patients with frequent or severe exacerbations are at an increased risk of further exacerbations, leading to a substantial healthcare burden associated with COPD [4, 5]. Chronic bronchitis (CB), which is classically defined as having chronic cough and sputum for at least 3 months for 2 consecutive years, is one of the strong risk factors of exacerbations [6–8].

For COPD patients with CB, bronchodilators with inhaled corticosteroids (ICSs) and/or phosphodiesterase-4 (PDE-4) inhibitors are currently recommended to reduce the frequency of exacerbations. However, COPD patients with a body mass index (BMI) less than 25 kg/m^2^ are at higher risk of pneumonia with ICS use [9, 10], and those with a lower BMI are also prone to discontinue PDE-4 inhibitor use due to adverse events [11], making the treatment maintenances more complicated. While this 25 kg/m^2^ BMI cut-off defines obesity in Asia–Pacific population, data from Asian countries showed that COPD patients with high BMI tend to have less symptom, better health-related quality of life and lung function, and fewer exacerbation and death, which suggests the obesity paradox [12–16].

Given that the BMI of Asian patients with COPD is consistently lower than that of Western patients [17], Asian patients with CB phenotype may have a different BMI distribution and a different impact of BMI on acute exacerbation compared with Western patients. We therefore conducted a multicenter cohort study to investigate the association between BMI and COPD exacerbations among Korean COPD patients with CB, and further compared these results with data from the Genetic Epidemiology of COPD (COPDGene) study. We hypothesized that Korean patients with CB phenotype and BMI < 25 kg/m^2^ would have higher risk of COPD exacerbation than other patient groups.

## Methods

### Study population and design

The Korean COPD Subgroup Study (KOCOSS) is an ongoing, multicenter observational cohort study, which has recruited COPD patients from referral hospitals in South Korea since December 2011 [18]. Patients who were diagnosed with COPD by a pulmonologist, aged ≥ 40 years, had a post-bronchodilator forced expiratory volume in 1 s/forced vital capacity (FEV_1_/FVC) < 0.7, and showed respiratory symptoms were included. Detailed sociodemographic data, medical histories, questionnaires including the COPD assessment test (CAT) and the COPD-specific St. George’s Respiratory Questionnaire (SGRQ-C), and results of laboratory tests, pulmonary function tests, and imaging tests were prospectively collected. This study was approved by the Institutional Review Board of each participating hospitals and written informed consent was obtained from all patients.

The COPDGene study is an observational, multicenter, longitudinal analysis of > 10,000 subjects with at least a 10 pack-year smoking history, with and without COPD [19, 20]. For the comparison with the KOCOSS, we only used the data of 4479 patients with a post-bronchodilator FEV_1_/FVC < 0.7 from phase I of the COPDGene study (from January 2008 to July 2011).

### Chronic bronchitis and BMI

In this study, CB was defined using the SGRQ-C, when subjects responded “almost every day” or “most days a week” to both of the questions: “Over the last 4 weeks, I have coughed” and “Over the last 4 weeks, I have brought up phlegm (sputum)”. Despite the relatively short-term period assessment, this alternative definition using SGRQ has been shown to identify more subjects with CB phenotype, who share strikingly similar clinical and radiologic characteristics with the classically defined CB patients [21, 22]. We used a BMI cut-off of 25 kg/m^2^, which is known as the risk factor for pneumonia development with ICS use [9, 10]. Study participants were categorized into four groups: non-CB with BMI ≥ 25 kg/m^2^, non-CB with BMI < 25 kg/m^2^, CB with BMI ≥ 25 kg/m^2^, and CB with BMI < 25 kg/m^2^.

### Acute exacerbation of COPD

The primary outcome of our analysis was the development of acute exacerbations during a 1-year follow-up. We assessed acute exacerbations based on claims data obtained from the Korean Health Insurance Review and Assessment Service (HIRA) database, by merging them with the KOCOSS data. Moderate exacerbations were defined as outpatient visits with an ICD-10 COPD code (J43.x−44.x, except J430, as the primary or within the fifth secondary diagnosis) and the prescription of systemic steroids with or without antibiotics. Severe exacerbations were defined as emergency department visits or hospitalization with an ICD-10 COPD code and the prescription of systemic steroids with or without antibiotics [23]. In the COPDGene cohort, exacerbations were self-reported using standardized questionnaires, at baseline and every 3 to 6 months during follow-up [24].

### Statistical analyses

Chi-square tests or one-way analysis of variance for normally distributed data and the Kruskal–Wallis test for skewed data were used to identify differences in demographic and clinical factors among the four patient groups at baseline. To evaluate the risk of acute exacerbations of COPD during the 1-year follow-up, zero-inflated Poisson regression were applied, in which exacerbations were modeled continuously as the number of exacerbations over the 1st year after enrollment in the KOCOSS, with incidence rate ratios (IRRs) and 95% CIs. Considering the outcome with excess zero, Poisson, zero-inflated Poisson, negative binominal, and zero-inflated negative binominal regressions were performed by modeling the count data of exacerbations. Based on the fit statistic of the Akaike Information Criterion (AIC) and Bayesian Information Criterion (BIC), the zero-inflated Poisson with smaller AIC and BIC values was selected. Multivariate regression analyses were also performed, using a set of different models: model 1 adjusted for age (years), sex, educational level (middle school or less vs. high school or more), smoking status (current smoker vs. ex-smoker vs. never-smoker), and post-bronchodilator FEV_1_ (continuous); model 2 further adjusted for use of ICS (yes vs. no), Charlson comorbidity index (continuous) and exacerbation history in the previous year (yes vs. no). For COPDGene cohort, exacerbations were dichotomized as either no exacerbation or any exacerbations over the 1st year after enrollment, and analyzed using logistic regression analysis, with odds ratios (ORs) and 95% confidence intervals (CIs). All P values were two sided, with statistical significance defined as P < 0.05. All statistical analyses were performed using SAS 9.4 (SAS Institute, Cary, NC).

## Results

### Study subjects

**Fig. 1:**
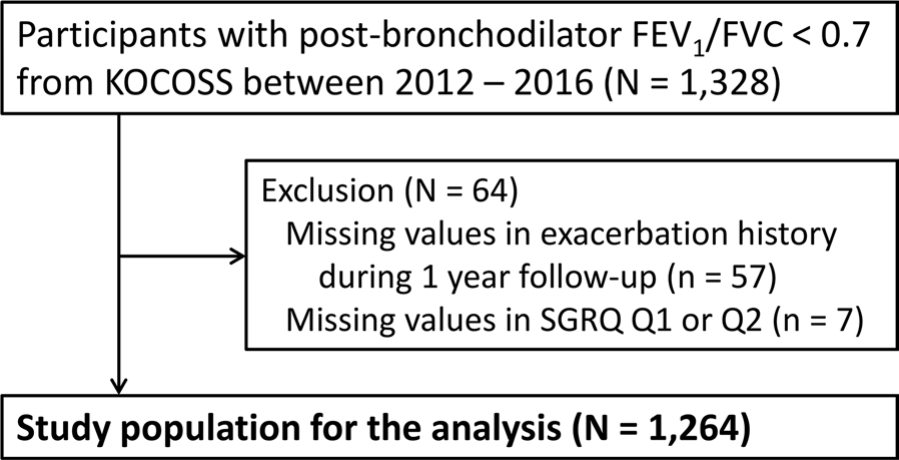
Flow diagram of the study population. *There were no missing values in BMI. BMI, body mass index; CB, chronic bronchitis; KOCOSS, Korean COPD Subgroup Study; SGRQ, St. George’s Respiratory Questionnaire

A total of 1328 subjects with spirometry-defined COPD were enrolled in the KOCOSS from January 2012 to December 2016. Those whose records contained missing values (n = 7) and those who were not linked to the HIRA database (n = 57) were excluded. Finally, 1264 patients were included in the analysis (Fig. 1). Mean (standard deviation [SD]) age was 69.1 (7.8) years, and most participants were men (91%). The majority of the patients were former (64.9%) or current (26%) smokers, while 9.1% were never smokers. Mean (SD) BMI was 22.9 (3.4) kg/m^2^ (Table 1).

**Table 1:** Baseline characteristics of patients with COPD from the KOCOSS according to the presence of chronic bronchitis and BMI categories

	Total (N = 1264)	Non-CB		CB		*P*
		BMI ≥ 25 (n = 230)	BMI < 25 (n = 583)	BMI ≥ 25 (n = 98)	BMI < 25 (n = 353)	
Age, years	69.1 ± 7.8	68.9 ± 7.4	69.9 ± 7.7	67.5 ± 7.9	68.3 ± 8.0	**0.003**
Sex, male	1150 (91.0)	200 (87.0)	536 (91.9)	89 (90.8)	325 (92.1)	0.095
Smoking status (N = 1260)						**0.002**
Current smoker	327 (26.0)	48 (20.9)	133 (23.0)	27 (27.6)	119 (33.7)	
Ex-smoker	818 (64.9)	153 (66.5)	391 (67.5)	63 (64.3)	211 (59.8)	
Never smoker	115 (9.1)	29 (12.6)	55 (9.5)	8 (8.2)	23 (6.5)	
Education (N = 1260)						0.118
Middle school or less	727 (57.7)	122 (53.0)	327 (56.4)	58 (59.2)	220 (62.5)	
High school or more	533 (42.3)	108 (47.0)	253 (43.6)	40 (40.8)	132 (37.5)	
Area of residence (N = 1223)						0.555
Urban area	481 (39.3)	144 (63.4)	338 (60.4)	51 (54.8)	209 (60.9)	
Rural area	742 (60.7)	83 (36.6)	222 (39.6)	42 (45.2)	134 (39.1)	
CAT score (N = 1253)	15.2 ± 7.8	12.8 ± 6.80	13.3 ± 7.05	16.6 ± 7.34	19.4 ± 8.07	**< 0.0001**
CAT score ≥ 10	931 (74.3)	151 (65.9)	387 (67.2)	81 (83.5)	312 (88.9)	**< 0.0001**
mMRC grade (N = 1263)	1.43 ± 0.90	1.26 ± 084	1.37 ± 0.91	1.43 ± 0.86	1.65 ± 0.90	**< 0.0001**
mMRC grade ≥ 2	495 (39.2)	72 (31.3)	209 (35.8)	40 (40.8)	174 (49.4)	**< 0.0001**
SGRQ-C (N = 1259)						
Symptom	44.6 ± 20.6	36.2 ± 16.0	37.2 ± 17.6	55.4 ± 19.5	59.4 ± 18.7	**< 0.0001**
Activity	46.1 ± 23.6	40.7 ± 21.0	44.0 ± 23.3	46.7 ± 22.5	53.1 ± 24.5	**< 0.0001**
Impact	24.4 ± 19.6	18.6 ± 16.4	20.5 ± 17.1	29.3 ± 20.2	33.3 ± 21.7	**< 0.0001**
Total	34.3 ± 18.9	28.1 ± 15.7	30.3 ± 17.2	38.8 ± 18.6	43.6 ± 20.0	**< 0.0001**
Exacerbation in the previous year (N = 1253)^a^	309 (24.7)	41 (17.9)	137 (23.7)	21 (21.6)	110 (31.4)	**0.002**
History of asthma (N = 1253)	473 (37.7)	86 (37.4)	224 (39.0)	32 (33.0)	131 (37.3)	0.722
Charlson comorbidity index (CCI)	2.13 ± 1.49	2.35 ± 1.42	2.16 ± 1.61	2.08 ± 1.28	1.94 ± 1.34	**0.010**
CCI ≥ 3	363 (28.7)	89 (38.7)	162 (27.8)	26 (26.5)	86 (24.4)	**0.002**

Data are presented as mean ± standard deviation or number (%). *P* values < 0.05 are presented in bold

BMI, body mass index; CAT, COPD assessment test; CB, chronic bronchitis; CCI, Charlson comorbidity index; COPD, chronic obstructive pulmonary disease; KOCOSS, Korean COPD Subgroup Study; mMRC, modified medical research council; SGRQ-C, COPD-specific St. George’s Respiratory Questionnaire

^a^Number (%) of the patients who experienced at least one moderate or severe acute exacerbation in the year prior to enrollment

### Distribution of BMI according to CB phenotype

In total, 451 (35.7%) patients had CB. Patients with CB had a significantly lower BMI than those without CB (22.5 [3.5] vs. 23.1 [3.3] kg/m^2^, P = 0.003). The prevalence of underweight (BMI < 18.5 kg/m^2^) patients were higher in CB group (13.5% vs. 8.4%, P = 0.002).

**Fig. 2:**
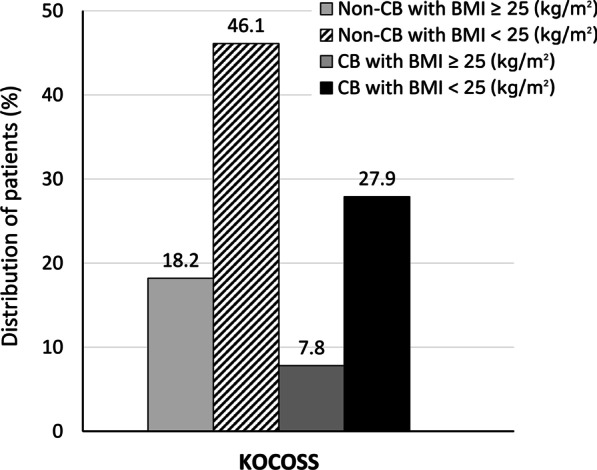
Distribution of patients according to the presence of chronic bronchitis and BMI categories. BMI, body mass index; CB, chronic bronchitis; KOCOSS, Korean COPD Subgroup Study

According to the presence of CB and a BMI cut-off values of 25 kg/m^2^, 230 (18.2%), 583 (46.1%), 98 (7.8%), and 353 (27.9%), patients were categorized into non-CB with BMI ≥ 25 kg/m^2^, non-CB with BMI < 25 kg/m^2^, CB with BMI ≥ 25 kg/m^2^, and CB with BMI < 25 kg/m^2^ groups, respectively (Fig. 2). Patients in the CB with BMI < 25 kg/m^2^ group were more likely to be current smokers and have dyspnea and higher CAT and SGRQ scores than patients in the other groups. CB with BMI < 25 kg/m^2^ group also had the highest exacerbation history (31.4%) in the previous year among four groups (Table 1).

Baseline pulmonary function test results are shown in Table 2. Compared with patients with a BMI ≥ 25 kg/m^2^, those with a BMI < 25 kg/m^2^ had significantly lower FEV_1_, regardless of CB. In addition, patients in the CB with BMI < 25 kg/m^2^ group had a lower diffusing capacity for carbon monoxide (DLco) and more hyperinflation than those in the other groups. At baseline, about half (51.5%) of patients in the CB with BMI < 25 kg/m^2^ group were receiving ICS-containing therapy, as triple therapy in most cases.

**Table 2:** Baseline pulmonary function parameters and maintenance medications of patients with COPD from the KOCOSS according to the presence of chronic bronchitis and BMI categories

	Total (N = 1264)	Non-CB		CB		*P*
		BMI ≥ 25 (n = 230)	BMI < 25 (n = 583)	BMI ≥ 25 (n = 98)	BMI < 25 (n = 353)	
Pulmonary function						
Post bronchodilator FVC, L	3.16 ± 0.82	3.14 ± 0.84	3.12 ± 0.82	3.35 ± 0.74	3.20 ± 0.82	**0.046**
Post bronchodilator FVC, % pred (N = 1262)	84.1 ± 18.6	82.4 ± 18.9	83.7 ± 17.8	84.3 ± 15.1	85.6 ± 20.4	0.203
Post bronchodilator FEV_1_, L	1.61 ± 0.57	1.76 ± 0.56	1.57 ± 0.58	1.82 ± 0.52	1.51 ± 0.53	**< 0.0001**
Post bronchodilator FEV_1_, % pred (N = 1262)	60.8 ± 19.6	66.2 ± 18.8	60.1 ± 20.0	65.4 ± 15.9	57.1 ± 19.4	**< 0.0001**
≥ 80% pred	181 (14.3)	46 (20.0)	83 (14.3)	16 (16.5)	36 (10.2)	**< 0.0001**
50% pred ≤ FEV_1_ < 80% pred	704 (55.8)	146 (63.5)	302 (51.9)	68 (70.1)	188 (53.3)	
< 50% pred	377 (29.9)	38 (16.5)	197 (33.8)	13 (13.4)	129 (36.5)	
Post bronchodilator FEV_1_/FVC	0.51 ± 0.13	0.56 ± 0.11	0.51 ± 0.14	0.54 ± 0.11	0.47 ± 0.12	**< 0.0001**
Bronchodilator response^a^	96 (7.6)	16 (7.0)	32 (5.5)	10 (10.2)	38 (10.8)	**0.020**
Diffusing capacity, % pred (N = 970)	75.3 ± 23.8	82.3 ± 21.2	76.1 ± 25.3	79.2 ± 21.9	68.4 ± 21.7	**< 0.0001**
< 60% pred	240 (24.7)	20 (11.3)	114 (26.0)	14 (17.3)	92 (33.6)	**< 0.0001**
RV/TLC, % (N = 754)	45.0 ± 12.8	43.5 ± 11.5	46.2 ± 13.82	40.4 ± 12.4	45.4 ± 11.8	**0.005**
6MWD, m (N = 1003)	374.8 ± 116.5	379.9 ± 110.5	379.8 ± 118.9	388.8 ± 106.9	360.0 ± 118.0	0.071
Baseline medication						
No	116(9.2)	27(11.7)	56(9.6)	7(7.1)	26(7.4)	**0.282**
Yes	1148(90.8)	203(88.3)	527(90.4)	91(92.9)	327(92.6)	
LAMA (N = 1124)	744 (66.2)	124 (61.7)	334 (64.0)	56 (66.7)	230 (72.6)	**0.034**
LABA (N = 1063)	207 (19.5)	31 (16.4)	96 (19.5)	16 (20.5)	64 (21.1)	0.633
ICS + LABA (N = 1082)	504 (46.6)	77 (38.7)	229 (45.9)	39 (52.0)	159 (51.5)	**0.031**
ICS + LABA + LAMA (N = 1058)	395 (37.3)	56 (29.3)	191 (39.1)	20 (28.2)	128 (41.7)	**0.012**
PDE-4 inhibitor (N = 1022)	57(5.6)	7(3.8)	23(4.9)	1(1.4)	26(8.7)	**0.023**

Data are presented as mean ± standard deviation or number (%). *﻿P* values < 0.05 are presented in bold

BMI, body mass index; CB, chronic bronchitis; COPD, chronic obstructive pulmonary disease; ICS, inhaled corticosteroid; FEV_1_, forced expiratory volume in 1 s; FVC, forced vital capacity; KOCOSS, Korean COPD Subgroup Study; LABA, long-acting beta-2 agonist; LAMA, long-acting muscarinic antagonist; PDE-4 inhibitor, phosphodiesterase-4 inhibitor; RV, residual volume; TLC, total lung capacity; 6MWD, 6-min walk distance

^a^Positive bronchodilator response was defined as the post-bronchodilator increase in FEV_1_ or FVC of at least 12% and 200 mL from baseline values at 15 min after inhalation of 400 μg of salbutamol

### Risk of COPD acute exacerbations during 1-year follow-up

During the 1-year follow-up, COPD exacerbations, defined as at least one moderate or severe exacerbation, developed in 76 (33.0%), 251 (43.1%), 40 (40.8%), and 184 (52.1%) patients in the non-CB with BMI ≥ 25 kg/m^2^, non-CB with BMI < 25 kg/m^2^, CB with BMI ≥ 25 kg/m^2^, and CB with BMI < 25 kg/m^2^ groups, respectively. The unadjusted IRR for exacerbations associated with CB was 1.36 (95% CI 1.14–1.62), while that associated with a BMI < 25 kg/m^2^ was 1.46 (95% CI 1.19–1.79). Compared with that in the non-CB with BMI ≥ 25 kg/m^2^ group, the unadjusted IRR (95% CI) for exacerbations was 1.43 (1.10–1.84), 1.32 (0.90–1.94), and 1.90 (1.45–2.49) in the non-CB with BMI < 25 kg/m^2^, CB with BMI ≥ 25 kg/m^2^, and CB with BMI < 25 kg/m^2^ groups, respectively. We adjusted for potential confounders including age, sex, smoking status, FEV_1_, ICS use, and exacerbation history in the previous year. In the fully adjusted model, the risk showed an increasing trend across the non-CB with BMI < 25 kg/m^2^, CB with BMI ≥ 25 kg/m^2^, and CB with BMI < 25 kg/m^2^ groups (adjusted IRR [95% CI] 1.21 [0.89–1.62], 1.20 [0.77–1.88], and 1.41 [1.02–1.91], respectively, compared to the non-CB with BMI ≥ 25 kg/m^2^ group) (Table 3). There was no interaction between the presence of CB and BMI categories.

**Table 3:** Incidence and the risk ratios of COPD exacerbation (≥ 1 moderate or ≥ 1 severe) during 1-year follow-up according to the presence of chronic bronchitis and BMI categories in 1264 patients from the KOCOSS

	Cases/at risk	Person-years	Incidence rate (per 1000 person-years)	Crude IRR (95% CI)	Adjusted^a^ IRR (95% CI)	
					Model 1	Model 2
Chronic bronchitis						
Non-CB	327/813	629	520	*Reference*	*Reference*	*Reference*
CB	224/451	317	707	**1.36 (1.14–1.62)**	**1.33 (1.12–1.59)**	1.19 (0.97–1.44)
BMI (kg/m^2^)						
BMI ≥ 25	116/328	265	438	*Reference*	*Reference*	*Reference*
BMI < 25	435/936	681	639	**1.46 (1.19–1.79)**	1.20 (0.97–1.48)	1.23 (0.97–1.56)
Chronic bronchitis and BMI (kg/m^2^)						
Non-CB and BMI ≥ 25	76/230	189	402	*Reference*	*Reference*	*Reference*
Non-CB and BMI < 25	251/583	439	572	**1.43 (1.10–1.84)**	1.20 (0.89–1.55)	1.21 (0.89–1.62)
CB and BMI ≥ 25	40/98	76	526	1.32 (0.90–1.94)	1.36 (0.91–2.00)	1.20 (0.77–1.88)
CB and BMI < 25	184/353	241	763	**1.90 (1.45–2.49)**	**1.58 (1.19–2.10)**	**1.41 (1.02–1.91)**

﻿*P* values < 0.05 are presented in bold. BMI, body mass index; CB, chronic bronchitis; CCI, Charlson comorbidity index; CI, confidence interval; COPD, chronic obstructive pulmonary disease; FEV_1_, forced expiratory volume in 1 s; ICS, inhaled corticosteroids; IRR, incidence rate ratios

^a^Model 1 was adjusted for age, sex, educational level, smoking status (current smoker vs. ex-smoker vs. never-smoker) and post bronchodilator FEV_1_(continuous). Model 2 was further adjusted for ICS use (yes vs. no), CCI (continuous) and exacerbation in the previous year (yes vs. no) in addition to Model 1

### Comparison with COPDGene study

Using the same inclusion and exclusion criteria for KOCOSS cohort, 3979 non-Hispanic White or African American patients with COPD were included in the analysis. Despite differences in major demographics and clinical manifestations of patients from two countries, the prevalence of CB was very similar between two cohorts (35.7% in KOCOSS and 39.3% in COPDGene). However, using BMI cut-off value of 25 kg/m^2^, the proportion of CB with BMI < 25 kg/m^2^ group was only 13.4% in COPDGene study (Additional file 1: Fig. S1). In COPDGene study, patients with CB had a higher risk of exacerbations than those without CB (unadjusted OR [95% CI] 1.85 [1.57–2.18]). However, the development of acute exacerbations did not differ according to BMI categories in COPDGene study (Additional file 2: Fig. S2).

## Discussion

In the nationwide COPD cohort of South Korea, the majority of patients with CB had a BMI < 25 kg/m^2^ and the proportion of patients with both CB and BMI < 25 kg/m^2^ was 28% of all COPD patients. Compared with other patient groups, those in CB with BMI < 25 kg/m^2^ group had more dyspnea, a poorer quality of life, and a lower lung function at baseline. During the 1-year follow-up, the risk of COPD acute exacerbations was most pronounced in patients with CB with BMI < 25 kg/m^2^, even after adjustments for potential confounders. This study revealed this non-obese CB phenotype, who are not uncommon among Korean COPD patients, but at higher risk of COPD acute exacerbation, for whom more specific phenotype-driven approaches might be needed in clinical practice.

The prevalence of CB in this study was similar to that reported in the previous literature, ranging from 22 to 42% [25–27]. The presence of CB was associated with not only the previous exacerbation prior to enrollment, but also an increased risk of exacerbations during the 1-year follow-up. These findings are consistent with those of previous studies [7, 28, 29] and emphasize the importance of identifying patients with a CB phenotype and managing them optimally.

The first important finding of this study is the BMI distribution according to CB phenotype, which showed a significantly lower BMI in CB patients compared to those without CB. This has been previously reported in the study using KOCOSS cohort, which was irrespective of CB definition (classic, SGRQ, or CAT definition) [22]. However, this is clearly different from the studies conducted in Western populations, which found that patients with CB had a similar or even higher BMI than those without CB [8, 21, 28, 30, 31], recapitulating the classic concept of “blue bloaters” [32]. Although it is a common notion that Asian patients with COPD have lower BMIs than their Western counterparts [17, 33], our study adds that Asian COPD patients with a CB phenotype have an even lower BMI than those without a CB phenotype.

BMI was an important risk factor for developing exacerbations in the KOCOSS cohort. In previous studies, a low BMI, weight loss, or a low fat-free mass index (FFMI) have been consistently related to increased mortality in patients with COPD [34–37]. However, the association between a low BMI and the risk of COPD exacerbations remains unclear. The Copenhagen General Population Study showed a trend of an increasing exacerbation risk in subjects with a low BMI, which, however, did not reach statistical significance [38]. In the COPDGene study, obesity (BMI ≥ 30 kg/m^2^) was associated with an increased risk of severe exacerbations in a dose-dependent manner [39], reflecting a U-shaped relationship, which implies that overweight patients have the lowest risk [40]. Data from Asian countries showed that obese COPD patients tend to experience fewer exacerbations [13–15]. Accordingly, our findings further document that a BMI < 25 kg/m^2^ adversely influences exacerbation risk beyond the presence of CB in Asian patients. There are possible explanations for higher exacerbation risk in non-obese CB patients. First, compared with CB patients with BMI ≥ 25 kg/m^2^, those with BMI < 25 kg/m^2^ had lower DLco, suggesting that this group may have higher degree of emphysema, which might have affected the increased risk of exacerbation [41, 42]. Further study including chest CT parameters such as emphysema index is needed to better define the clinical phenotypes and validate our findings. Second, a high BMI might be a marker of a greater muscle mass or better nutritional status, which are associated with better outcome in COPD [35, 43, 44]. Third, CB patient with BMI ≥ 25 kg/m^2^ had lower residual volume/total lung capacity than those with BMI < 25 kg/m^2^, which is consistent with that higher BMI is associated with lesser hyperinflation (as lung volume decreases with increase in BMI) [45]. In addition to physical inactivity and deconditioning, hyperinflation has been associated with frequent exacerbation [46].

In this study, 28% of KOCOSS patients had both CB and a BMI < 25 kg/m^2^, and these patients were at the highest risk of exacerbations. The impact of a low BMI also extends to therapeutic choices for exacerbation prevention in Asian patients with COPD. ICS and PDE-4 inhibitors are representative medications recommended to reduce exacerbation in patients with frequent exacerbations. However, these recommendations are based on data primarily from Western populations. Indeed, prolonged use of ICS has been associated with an increased risk of pneumonia in patients with BMI < 25 kg/m^2^ [9, 10]. Similarly, the reactivation of tuberculosis (TB) may complicate ICS treatment in Asian populations with a TB burden, as a low BMI is a notable risk factor for TB [47]. The rate of PDE-4 inhibitor discontinuation due to adverse events was also higher in Korean patients than in patients from Western countries, and this was especially observed in those with low BMI [11]. Taken together, Asian COPD patients with CB distinctly have a lower BMI than those without CB, which increases their risk of exacerbations as well as of potential difficulties in maintaining treatment. Further studies are therefore needed to better understand this specific patient population and to set guidelines for the optimal treatment.

This study has some potential limitations. First, the KOCOSS study recruited patients with COPD from pulmonology clinics in Korea, which limits the generalizability of the findings to the entire COPD population of Asia. Second, the BMI cut-off of 25 kg/m^2^ applied to our Korean population might be too high to define a low BMI [48]. Instead, it is more appropriate to interpret this group with BMI < 25 kg/m^2^ as “non-obese CB patients”. However, when we repeated the main analysis of the KOCOSS cohort using a 23-kg/m^2^ cut-off (upper normal weight for Asian populations by the WHO [12]), we obtained similar results (Additional file 3: Table S1). We therefore decided to use a cut-off of 25 kg/m^2^ to emphasize that while a considerable proportion of Asian patients with COPD is “non-obese CB phenotype” who are at a high risk of exacerbations and adverse event following treatment, there is a lack of treatment guidelines for this population. Third, we do not have other measures, such as FFMI using bioelectrical impedance or dual-energy X-ray absorption, or mid-thigh muscle cross-sectional area and visceral adipose tissue accumulation on CT, which better represent body composition. Given that cachexia (based on consensus definition [37]), a low FFMI, or multi-organ loss of tissue have been shown to be more predictive of mortality in COPD [37, 42], our findings based on BMI alone need to be validated in further studies using more comprehensive measurements of anthropometry and adiposity. Finally, despite using identical definitions for CB and BMI cut-off values, measurements for exacerbations differed between the two cohorts. As the KOCOSS defined exacerbations using claims data, the absolute rate of exacerbations was higher than that reported in the COPDGene study, which was based on self-report questionnaires. However, we only compared the relative risks of groups within each cohort separately.

Using multicenter COPD cohorts from South Korea and the United States, we report that Korean COPD patients with a CB phenotype have a lower BMI than those without a CB phenotype, while no such difference in BMI was observed among patients in the COPDGene study. In Korean COPD patients, having both CB and a BMI < 25 kg/m^2^ was associated with an increased risk of exacerbations even after adjustments for potential confounders, whereas exacerbation risk did not differ according to BMI in patients from the COPDGene study. Considering that a BMI < 25 kg/m^2^ often limits treatment options preventing exacerbations, more medical attention and modified guidelines are needed for non-obese CB patients in Asia.

## Supplementary Information

**Additional file 1: Figure S1.** Distribution of patients according to the presence of chronic bronchitis and BMI categories. BMI, body mass index; CB, chronic bronchitis; COPDGene study, COPD Genetic Epidemiology study.

**Additional file 2: Figure S2. **The risk of COPD exacerbation* during 1-year follow-up according to the presence of chronic bronchitis and BMI categories among 3979 patients from the COPDGene study. *Exacerbation was defined as at least one mild, moderate or severe exacerbation in the COPDGene study. BMI, body mass index; CB, chronic bronchitis; COPD Genetic Epidemiology study.

**Additional file 3: Table S1.** Incidence and the risk ratios of COPD exacerbation (≥ 1 moderate or ≥ 1 severe) during 1-year follow-up according to the presence of chronic bronchitis and BMI categories (using cut-off 23 kg/m^2^) in 1264 patients from the KOCOSS.

## Data Availability

The KOCOSS datasets used and analyzed in this study are available from the corresponding author upon reasonable request.
