# Supplementary figures and images for: Association of body mass index and COPD exacerbation among patients with chronic bronchitis
Source: Respir Res. 2022 Mar 7;23:52. doi: 10.1186/s12931-022-01957-3 (PMC8900381; doi:10.1186/s12931-022-01957-3)

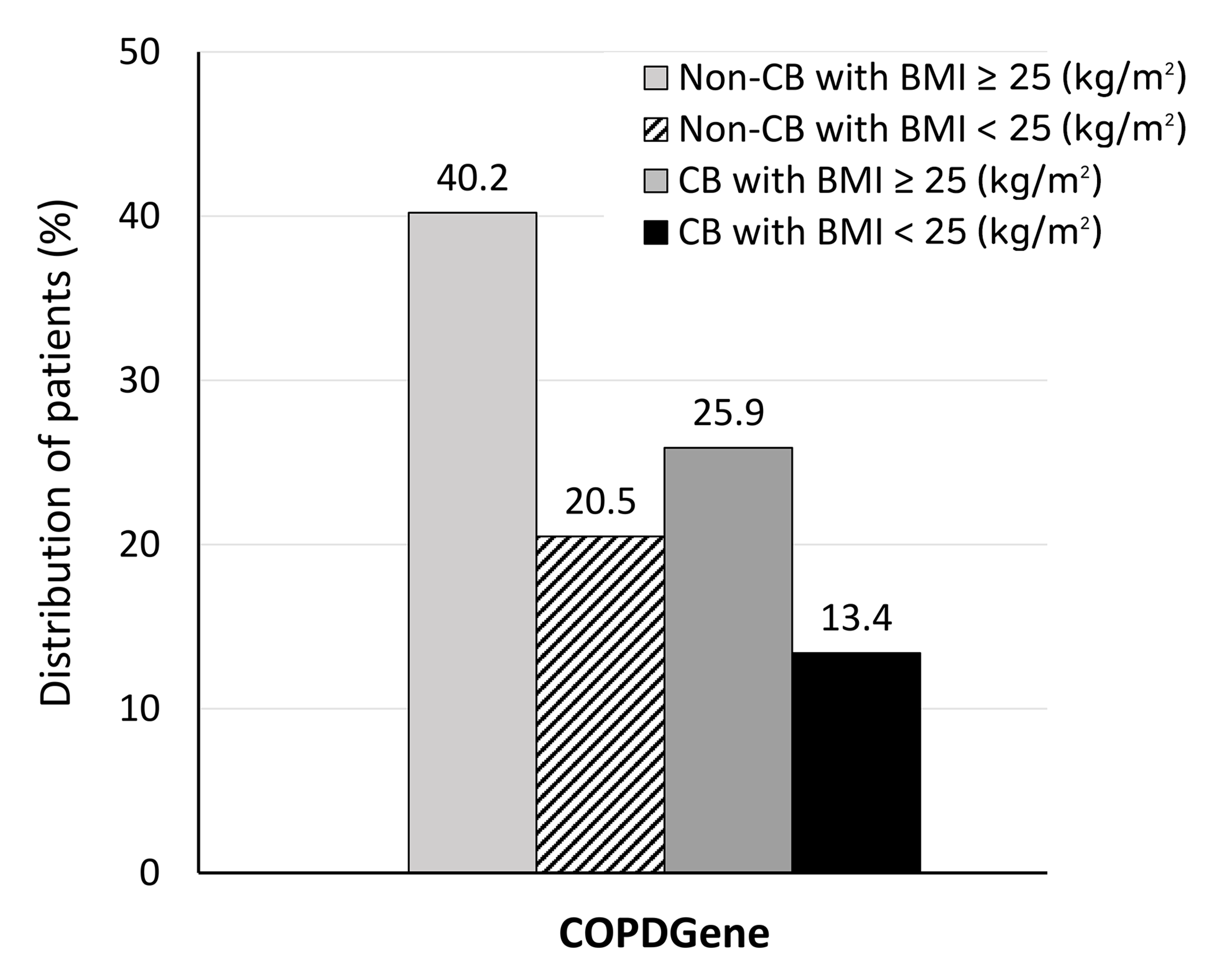

Supplement: Supplementary file 1 — Additional file 1: Figure S1. Distribution of patients according to the presence of chronic bronchitis and BMI categories. BMI, body mass index; CB, chronic bronchitis; COPDGene study, COPD Genetic Epidemiology study. [file 12931_2022_1957_MOESM1_ESM.tif]

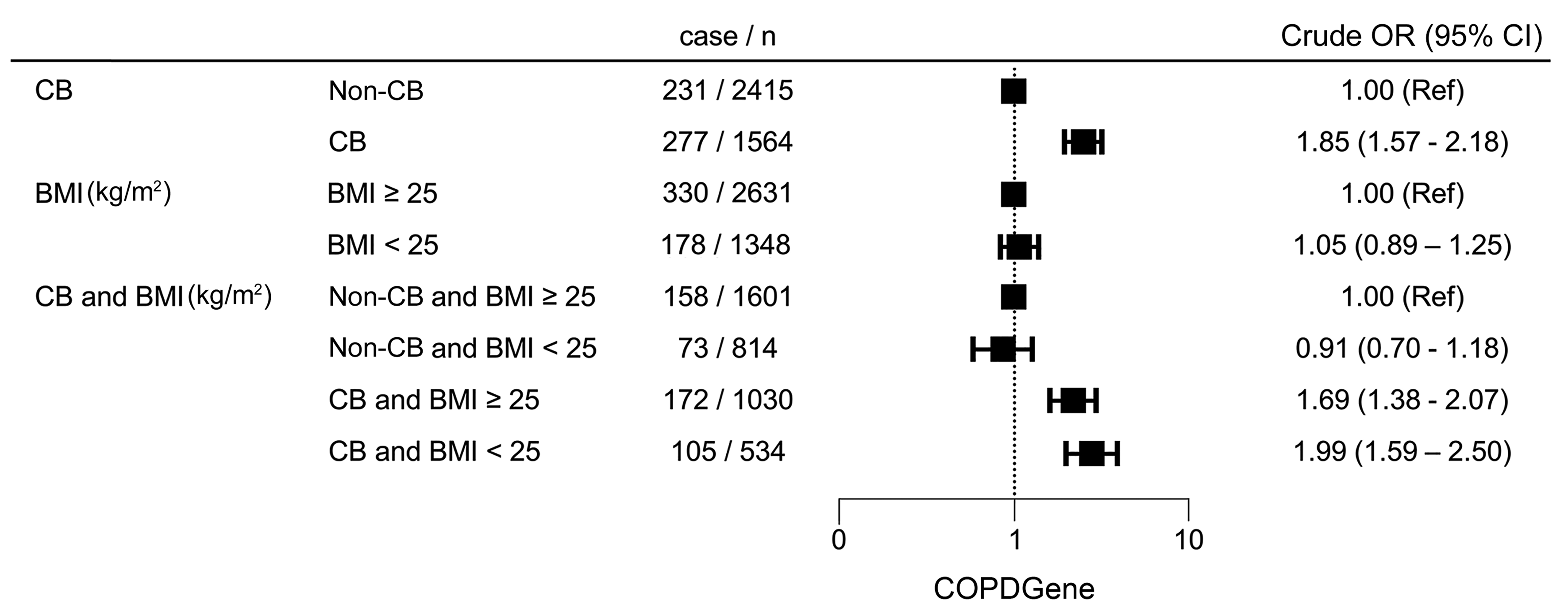

Supplement: Supplementary file 2 — Additional file 2: Figure S2. The risk of COPD exacerbation* during 1-year follow-up according to the presence of chronic bronchitis and BMI categories among 3979 patients from the COPDGene study. *Exacerbation was defined as at least one mild, moderate or severe exacerbation in the COPDGene study. BMI, body mass index; CB, chronic bronchitis; COPD Genetic Epidemiology study. [file 12931_2022_1957_MOESM2_ESM.tif]
