# Supplementary material for: Association of body mass index and COPD exacerbation among patients with chronic bronchitis
Source: Respir Res. 2022 Mar 7;23:52. doi: 10.1186/s12931-022-01957-3 (PMC8900381; doi:10.1186/s12931-022-01957-3)
Supplement: Supplementary file 3 — Additional file 3: Table S1. Incidence and the risk ratios of COPD exacerbation (≥ 1 moderate or ≥ 1 severe) during 1-year follow-up according to the presence of chronic bronchitis and BMI categories (using cut-off 23 kg/m2) in 1264 patients from the KOCOSS. [file 12931_2022_1957_MOESM3_ESM.docx]

**Table S1.** Incidence and the risk ratios of COPD exacerbation (≥1 moderate or ≥1 severe) during 1-year follow-up according to the presence of chronic bronchitis and BMI categories (using cut-off 23 kg/m^2^) in 1,264 patients from the KOCOSS

|  | **cases/at risk** | **Person- years** | **Incidence rate**  **(per 1,000 person-years)** | **Crude IRR**  **(95% CI)** | **Adjusted^*^ IRR (95% CI)** | |
| --- | --- | --- | --- | --- | --- | --- |
|  |  |  |  |  | **Model 1** | **Model 2** |
| **Chronic bronchitis** |  |  |  |  |  |  |
| Non-CB | 327/813 | 629 | 520 | *Reference* | *Reference* | *Reference* |
| CB | 224/451 | 317 | 707 | **1.36(1.14-1.62)** | **1.33 (1.12-1.59)** | 1.19 (0.97-1.44) |
| **BMI (kg/m^2^)** |  |  |  |  |  |  |
| BMI ≥ 23 | 228/593 | 467 | 488 | *Reference* | *Reference* | *Reference* |
| BMI < 23 | 323/671 | 479 | 675 | **1.38(1.16-1.64)** | 1.15(0.93-1.37) | 1.17(0.96-1.42) |
| **Chronic bronchitis and BMI (kg/m^2^)** |  |  |  |  |  |  |
| Non-CB and BMI ≥ 23 | 139/400 | 325 | 427 | *Reference* | *Reference* | *Reference* |
| Non-CB and BMI < 23 | 188/413 | 303 | 620 | **1.45(1.16-1.81)** | 1.23(0.97-1.54) | 1.25(0.97-1.63) |
| CB and BMI ≥ 23 | 89/193 | 141 | 629 | **1.47(1.13-1.93)** | **1.52(1.16-1.99)** | **1.37(1.02-1.87)** |
| CB and BMI < 23 | 135/258 | 175 | 770 | **1.80(1.42-2.29)** | **1.50(1.17-1.93)** | **1.34(1.01-1.79)** |

^*^ Model 1 was adjusted for age, sex, educational level, smoking status (current smoker vs. ex-smoker vs. never-smoker) and post bronchodilator FEV_1_(continuous).
Model 2 was further adjusted for ICS use (yes vs. no), CCI (continuous) and exacerbation in the previous year (yes vs. no) in addition to Model 1.

BMI, body mass index; CB, chronic bronchitis; CCI, Charlson comorbidity index; CI, confidence interval; COPD, chronic obstructive pulmonary disease; FEV_1_, forced expiratory volume in 1 second; ICS, inhaled corticosteroids; IRR, incidence rate ratios.
